# 

**Published:** 1899-12-16

**Authors:** 


					THE HOSPITAL. 41 The standard of highest purity,"?Lancet December 16tii, 1899.
> CADBURY'S r# U 15* CADBURY'S
COCOA jk COCOA
. ....         MO npnni HD AT VAT TT70 ITODn
ABSOLUTELY PURR. ^ DRUG! OR ALKALIES USED.
as corf Western Infirmary
No. 690.1 Vol. XXVII. [S^eadpear:] Saturday,
Dec. 16th, 1899.
[Subscription Terms,J pRICE TWOPENCE.

				

